# Psychometric Properties of Digital Divide Measurement Instruments in Health Contexts: Systematic Review

**DOI:** 10.2196/82005

**Published:** 2026-04-22

**Authors:** Noelia Navas-Echazarreta, Elena Chover-Sierra, César Rubio-Tarazón, Raquel María Martínez-Pascual, Pablo Del Pozo-Herce, Jesús Martínez-Tofé, Raúl Juárez-Vela, Antonio Martínez-Sabater

**Affiliations:** 1 Research Group GRUPAC, Pre-departmental Biomedical Sciences and Health Specialties Unit, Faculty of Health Sciences, University of La Rioja, C/ Duquesa de la Victoria, 88, Logroño, 26004, Spain, 941299059; 2 Nursing Care and Education Research Group (GRIECE), GIUV2019-456, Nursing Department, University of Valencia, Valencia, Spain; 3 Internal Medicine Department, Consorci Hospital General Universitari de Valencia, Valencia, Spain; 4 Research Group on Innovation in Health Care and Nursing Education (INcUidE), University of UNIE, Madrid, Spain; 5 Care Research Group (INCLIVA), University Clinical Hospital of Valencia, Valencia, Spain

**Keywords:** digital divide, psychometric properties, measurement instruments, digital health, health inequalities, systematic review, nursing

## Abstract

**Background:**

The digital divide constitutes a growing form of social inequality, influencing access to, use of, and benefit from digital health technologies. Accurate assessment of this phenomenon requires psychometrically sound measurement instruments, especially in health care contexts.

**Objective:**

This systematic review aimed to identify and evaluate the psychometric properties of instruments developed to measure the digital divide, with a particular focus on their application in health-related settings.

**Methods:**

A systematic review was conducted following the PRISMA (Preferred Reporting Items for Systematic Reviews and Meta-Analyses) 2020 and COSMIN (Consensus-Based Standards for the Selection of Health Measurement Instruments) guidelines. Literature searches were performed in PubMed, Scopus, Dialnet, and Web of Science for studies published between 2015 and 2025. Inclusion criteria required studies to report a psychometric evaluation of instruments measuring the digital divide.

**Results:**

Four studies met the inclusion criteria (ranging from 875 to 1337 participants). The identified scales primarily measured digital skills and capital. All studies reported adequate internal consistency, with Cronbach α coefficients ranging from 0.70 to 0.94. Structural validity was confirmed through factor analysis in all studies. However, data on temporal stability and responsiveness were absent.

**Conclusions:**

Existing instruments show potential for assessing digital inequality in health, but further psychometric validation and cultural adaptation are required. Nursing professionals are encouraged to lead efforts in developing and validating context-sensitive instruments to promote digital health equity.

## Introduction

Digital transformation has profoundly altered the way in which individuals interact with information, communication, and self-management of their health [1]. However, this evolution has not been uniform across all citizens. While some have benefited from these new digital tools, others have been left behind. This inequality is known as the digital divide, a multidimensional phenomenon encompassing disparities in access, use, digital skills, and benefits derived from technology [2].

This multidimensional concept involves not only inequalities in access to devices or connectivity (first-level divide) but also limitations in the competencies required to use technologies effectively (second-level divide) and disparities in the actual benefits obtained, such as improvements in health outcomes (third-level divide) [2,3]. Consequently, the digital divide acts as an emerging social determinant, disproportionately affecting older adults, individuals with low educational attainment, socioeconomically disadvantaged groups, and those with limited digital literacy [4,5].

Within the health care sector, the digital divide compromises access to digital services such as telemedicine, mobile health apps, self-management tools, and eHealth resources. This situation hinders the implementation of technology-based interventions and limits the effectiveness of public policies aimed at promoting equity [6-8]. These limitations particularly impact older adults, individuals with lower educational levels, those in vulnerable socioeconomic situations, and individuals with cognitive impairments [5]. This digital gap critically undermines the efficacy of telehealth interventions as patients lacking necessary digital skills or capital cannot effectively engage with virtual care models, thereby exacerbating existing health inequities [4,5,7].

In this regard, the COVID-19 pandemic exacerbated exclusion when a substantial portion of health care services transitioned to virtual environments. These difficulties in health care delivery were particularly evident among groups with low digital literacy or communication disabilities [6].

Despite the growing interest in this phenomenon, the measurement of the digital divide remains a challenge. Although scales and instruments assessing digital literacy or technological competencies exist, their specific psychometric validity, comprehensive structural approach, and cross-cultural applicability are not always ensured [7,8]. This situation undermines comparability across studies, limits accurate assessment of disparities, and hampers the design of evidence-based interventions [9].

Research conducted by Kayser et al [5] and Mota [10] relied on instruments focused on assessing digital literacy, attitudes, or knowledge regarding technology; however, these tools fail to adequately address the structural dimensions of the digital divide. Moreover, in several studies, such as that by Jongebloed et al [7], questionnaires lacking proper psychometric validation were used, thereby compromising the reliability of findings and restricting their utility in future research. Consequently, the guidelines proposed by Beaton et al [8] and the COSMIN (Consensus-Based Standards for the Selection of Health Measurement Instruments) checklist developed by Mokkink et al [9] are essential for the robust evaluation of measurement properties.

The availability of psychometrically sound instruments adapted to diverse sociocultural contexts is crucial to quantify the digital divide [5], understand its implications for health, and guide effective strategies for its reduction, particularly within nursing and public health, which play key roles in fostering digital equity.

In this context, this study aimed to conduct a systematic review of scientific literature to identify and analyze the psychometric properties of instruments designed to measure the digital divide, with special emphasis on their applicability in health care settings and their potential use in Spanish-speaking populations.

## Methods

### Study Design

This study was designed as a systematic review of scientific literature conducted in accordance with the PRISMA (Preferred Reporting Items for Systematic Reviews and Meta-Analyses) 2020 guidelines and the COSMIN framework [9,11] with the objective of comprehensively analyzing the scales used to measure the digital divide, with a particular focus on the health care context. The review protocol was registered in the PROSPERO database (registration number CRD420251068347).

The review focused on the structural and methodological dimensions of the instruments, as well as on their key psychometric properties, encompassing reliability, validity in all its forms (content, construct, convergent, discriminant, and predictive or concurrent), and structural fit assessed through exploratory factor analysis (EFA) and confirmatory factor analysis (CFA), in addition to the Rasch item response model [12].

The rigorous application of the PRISMA and COSMIN guidelines ensured process transparency, minimized methodological bias, and guaranteed quality in the selection and analysis of the relevant scientific literature [9,11].

### Search Strategy

The search strategy was designed to ensure a broad and exhaustive retrieval of scientific literature related to instruments measuring the digital divide.

A systematic search was conducted across 4 scientifically recognized databases—Scopus, Dialnet, PubMed, and Web of Science—aimed at covering both English-language literature and studies conducted in Spanish-speaking contexts. The databases were searched with the time window set from 2015 to 2025. The final search across all databases was executed on January 15, 2025.

Boolean search strings were constructed by combining controlled and free-text terms primarily in English. These terms were organized into 3 blocks interconnected using the Boolean operators “AND,” “OR,” and “NOT.” The first block referred to terms related to the digital divide (“digital divide” and “digital inequality”), the second referred to instruments or related tools (“scale,” “instrument,” and “tool”), and the third referred to the measurement of psychometric properties (“psychometric properties,” “validity,” and “measurement properties”).

Document retrieval and bibliographic management were performed using the RefWorks software (Clarivate Analytics). This information is summarized in Table 1, which details the databases searched, the specific Boolean strings applied, the total number of documents identified, and the number of documents ultimately selected for this research.

**Table 1.** The search strategy.

Database and search string	Documents retrieved, n	Documents selected, n
Scopus		
(“digital divide” or “digital inequality” or “digital gap”) and (“scale” or “instrument” or “measure” or “tool”) and (“psychometric Properties” or “validity” or “reliability” or “internal consistency” or “measurement properties”)	68	3
(“digital divide” or “digital inequality” or “digital gap”) and (“scale” Or “instrument”) and (“validity” or “reliability”)	35	1
Dialnet		
(“digital divide” or “digital inequality” or “digital gap”) and (“scale” or “instrument” or “measure” or “tool”) and (“psychometric Properties” or “validity” or “reliability” or “internal consistency” or “measurement properties”)	11	0
(“brecha digital” or “desigualdad digital”) and (“instrumento” or “escala” or “cuestionario”) and (“validez” or “fiabilidad” or “propiedades psicométricas”)	9	0
PubMed		
(“digital divide” or “digital inequality” or “digital gap”) and (“scale” or “instrument” or “measure” or “tool”) and (“psychometric Properties” or “validity” or “reliability” or “internal consistency” or “measurement properties”)	14	1
WOS^a^		
(“digital divide” or “digital inequality” or “digital gap”) and (“scale” or “instrument” or “measure” or “tool”) and (“psychometric Properties” or “validity” or “reliability” or “internal consistency” or “measurement properties”)	55	3
(“digital divide” or “digital gap”) and (“scale” or “measure”) and (“validity” or “reliability” or “psychometric properties”)	32	3

a WOS: Web of Science.

### Selection Criteria

The selection of studies included in this review was conducted through a careful, coherent, and progressive process. Studies published in English or Spanish, available in full text, and published within the time frame of 2015 to 2025 were included, coinciding with the increased research on the digital divide over the last decade, particularly following the acceleration of digitalization triggered by the COVID-19 pandemic.

Eligible articles were required to focus on the development, validation, adaptation, or application of structured instruments designed to measure the digital divide and provide data on their psychometric properties, including reliability, validity, internal consistency, or factor analysis.

Articles that did not directly address the measurement of the digital divide through psychometric instruments, as well as narrative reviews, opinion papers, and similar works, were excluded. Studies focusing exclusively on digital literacy without addressing digital inequalities or lacking psychometric data were also excluded. Selection was based on these inclusion and exclusion criteria following a detailed review of titles, abstracts, and full texts of articles meeting the eligibility requirements.

### Effect Measures

The psychometric properties of the identified scales were assessed using the COSMIN guidelines [9]. Effect measures included indicators of reliability (Cronbach α coefficient, ω coefficient, Kuder-Richardson Formula 20, and intraclass correlation coefficient [ICC]); content, construct, convergent, discriminant, and predictive or concurrent validity; factor analysis (EFA and CFA); and estimates derived from the Rasch item response model [12].

Unlike classical test theory, which focuses on total scores and sample-dependent statistics, item response theory (IRT) and Rasch modeling allow for the evaluation of individual item properties independent of the specific sample. Consequently, Rasch analysis is preferred for its ability to verify measurement invariance and transform ordinal data into linear interval measures, offering a more rigorous validation than classical test theory alone [9,12]. Each property was qualitatively classified according to the criteria by Terwee et al [13] as sufficient (+), insufficient (−), or indeterminate (?). This categorization facilitated the assessment of the methodological robustness of the instruments included in the review and supported the narrative synthesis of the findings [13].

### Study Selection Process

Studies were included if they were relevant to the research question, met the inclusion criteria, and demonstrated adequate methodological quality. Two reviewers (AM-S and CR-T) independently screened the titles and abstracts of retrieved documents to identify potentially eligible studies. The full texts of these studies were then independently assessed by 2 other reviewers (CR-T and NN-E). Discrepancies were resolved through discussion, with a third reviewer (AM-S) consulted when necessary. Interrater agreement was measured using the ICC.

### Study Selection and Data Extraction

Evaluated aspects included risk of bias, methodological design, and reporting quality. The PRISMA and COSMIN frameworks were applied to assess study quality. Data were identified, verified, and extracted independently by 2 authors (CR-T and NN-E). Extracted variables included author, year, country, instrument name, target population, number of items, reported psychometric properties, and applied statistical methods [9,11].

### Data Summarization

Results were synthesized narratively and organized into descriptive tables detailing the main characteristics, psychometric properties, and scientific quality of the identified instruments. Findings were grouped according to property type and corresponding evaluations.

## Results

### Overview

**Figure 1.**
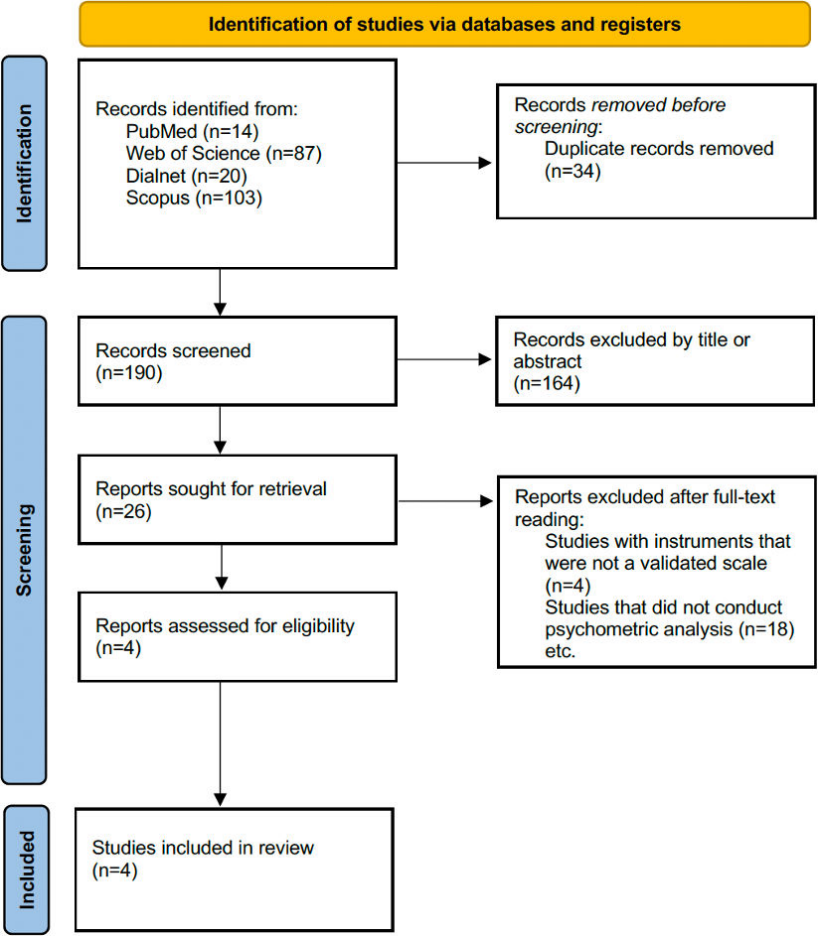
Selection process flow diagram.

Through a systematic bibliographic search across scientific databases, 224 records were initially identified. Of these 224 records, after removing 34 (15.2%) duplicates using RefWorks, 190 (84.8%) remained. Title and abstract screening resulted in the exclusion of 86.3% (164/190) of the remaining studies for not meeting the inclusion criteria. Consequently, 26 studies were selected for full-text review, of which 22 (88.5%) were excluded due to lack of instruments, absence of psychometric analysis, or limited thematic relevance. Ultimately, 4 studies met the inclusion criteria and were included in the systematic review, as illustrated in the PRISMA flow diagram (Figure 1). The ICC between reviewers was 0.80 (95% CI 0.68‐0.88).

### Studies Included

The 4 studies analyzed were published between 2015 and 2023. These investigations were conducted in the United Kingdom [14], the Netherlands [2,14], the United States [15], and Italy [16]. Two studies focused on samples of students or young individuals [2,15], whereas the remaining 2 administered questionnaires to representative adult populations [14,16]. The sample sizes in all included studies were adequate according to COSMIN standards (N>100), with respondent-to-item ratios ranging from 35 to 127, ensuring robust conditions for the factor analyses performed [2,14-16]. All included studies used a cross-sectional design.

### Identified Scales and Instruments

The 4 instruments evaluated different dimensions of the digital divide, as shown in Table 2. Each instrument was developed by the original authors and culturally adapted to the country where it was applied. The tools measured aspects such as digital competencies, digital capital, technological adaptability, and functional digital skills, with particular emphasis on the psychometric properties of the measurement tools used [2,14-16].

**Table 2.** Studies included in the systematic review.

Study	Study type	Measuring instrument	Language	Objective	Population	Cross-cultural adaptation
van Deursen et al [14], 2015	Cross-sectional study	ISS^a^	English	Develop a tested instrument for measuring internet skills	Adults from the UK^b^ and the Netherlands (N=1337)	No
van Deursen et al [2], 2022	Cross-sectional study	IoTSS^c^	English	Develop and validate a multidimensional scale of digital skills	Young Dutch people—15-25 y (N=875)	No
Puckett [15], 2022	Cross-sectional study	Digital Adaptability Scale	English	Measure digital adaptive capacity as a dimension of digital inequality	University students in the United States (N=897)	No
Addeo et al [16], 2023	Cross-sectional study	Digital Capital Index	Italian	Validate a composite index of digital capital in the original population	Italian adults (N=1016)	No

a ISS: Internet Skills Scale.

b UK: United Kingdom.

c IoTSS: Internet of Things Skills Scale.

The instruments were based on diverse theoretical frameworks, ranging from models focused on the collection and storage of digital resources [16] to self-assessment scales of performance in technological contexts [15] and European regulatory frameworks on fair competition [2]. All were administered through online surveys and underwent psychometric analysis.

### Psychometric Properties Assessed

Table 3 presents the analysis of the psychometric properties of the instruments identified in the 4 studies included in this review. Regarding factor analysis, Puckett [15] applied a 1-stage EFA, whereas van Deursen et al [14], Addeo et al [16], and van Deursen et al [2] conducted both EFA and CFA to verify the internal structure of their scales.

**Table 3.** Psychometric properties of the measurement instruments included in the review.

Study	Measuring instrument	Items, n	Reliability, Cronbach α	Factor analysis	Reported validity
van Deursen et al [14], 2015	ISS^a^	35	0.83-0.94	EFA^b^+CFA^c^	Content and construct validity
van Deursen et al [2], 2022	IoTSS^d^	25	0.89-0.92	EFA+CFA	Construct validity: convergent and discriminant
Puckett [15], 2022	Digital Adaptability Scale	12	0.77	EFA	Construct validity: convergent and discriminant
Addeo et al [16], 2023	Digital Capital Index	8	0.701-0.946	EFA	Content and construct validity

a ISS: Internet Skills Scale.

b EFA: exploratory factor analysis.

c CFA: confirmatory factor analysis.

d IoTSS: Internet of Things Skills Scale.

All studies reported Cronbach α values exceeding 0.70, indicating at least adequate internal consistency. This suggests that the items in the questionnaires consistently measured the same construct. However, it should be noted that none of the studies reported data on temporal stability (test-retest reliability) or interrater reliability, which limits the assessment of the instruments’ consistency over time [2,14-16].

Validity analysis demonstrated that the instruments accurately measured the intended concepts. van Deursen et al [14] and Addeo et al [16] assessed both construct and content validity, whereas Puckett [15] and van Deursen et al [2] examined convergent validity (similarity to previous tests) and discriminant validity (ensuring that the test does not measure unrelated concepts).

In reviewing the instruments used to assess digital skills and thereby measure the digital divide, 4 scales with robust psychometric evidence were identified in the included studies. The Digital Adaptability Scale [15] and the Digital Capital Index [16] were developed as new instruments, whereas the Internet of Things Skills Scale (IoTSS) [2] sought to validate its structure based on the preexisting Internet Skills Scale (ISS) [14] and additional items related to the Internet of Things.

The internal consistency reliability of the instruments was assessed using the Cronbach α coefficient. The interpretative thresholds were as follows: excellent (Cronbach α≥0.90), good (Cronbach α=0.80‐0.89), acceptable (Cronbach α=0.70‐0.79), moderate (Cronbach α=0.60‐0.69), poor (Cronbach α=0.50‐0.59), and unacceptable (Cronbach α<0.50) [17]. These thresholds align with established psychometric standards for the evaluation of internal consistency in health measurement instruments.

The factor structure of the instruments was analyzed using 2 primary approaches. First, EFA was used in studies where no a priori theoretical structure was assumed. This method is typically used during the development of new instruments or when the dimensionality of the construct is unknown. Regarding CFA, it was used in studies with a predefined hypothesis about the number of factors and the item-factor relationships based on existing theory or prior empirical evidence. Some studies applied a combined approach, initially exploring the underlying structure and subsequently confirming it in a separate analysis or sample [9].

The ISS [14], consisting of 35 items, demonstrated high internal consistency (Cronbach α=0.83‐0.94) and underwent both EFA and CFA, providing evidence of content and construct validity. Subsequently, the IoTSS [2], comprising 25 items with similarly elevated reliability coefficients (Cronbach α=0.89‐0.92), was validated through EFA and CFA, with construct validity specifically supported by convergent and discriminant evidence. Similarly, the Digital Adaptability Scale [15], consisting of 12 items and yielding a Cronbach α of 0.77, was validated via EFA, demonstrating construct validity through its convergent and discriminant relationships with other variables. Finally, the Digital Capital Index [16], which includes 8 items, exhibited a wide range in internal consistency (Cronbach α=0.701‐0.946) as a result of a 2-stage EFA and provided evidence of both content and construct validity. Collectively, these instruments exhibit adequate levels of reliability and validity, making them valuable tools for measuring digital competencies in various contexts.

### Methodological Quality Assessment

The risk of bias of the included studies was assessed using the COSMIN Risk of Bias tool, which evaluates the methodological rigor in the design, development, and application of measurement instruments (Table 4). Overall, the studies appropriately defined the construct to be measured and justified its theoretical relevance. However, recurring weaknesses were observed in the preliminary phases, particularly regarding the design of pilot-testing and the evaluation of item comprehensibility from the perspective of the target population.

**Table 4.** Assessment of the level of bias in tool design according to the COSMIN (Consensus-Based Standards for the Selection of Health Measurement Instruments) Risk of Bias tool.

Study	PROM^a^ (design)	PROM relevance and comprehensiveness	Pilot test design	Comprehensibility of the pilot test	Comprehensiveness of the pilot test	Final assessment (the lowest qualitative score obtained)
van Deursen et al [14], 2015	Very good	Very good	Very good	Very good	Very good	Very good
van Deursen et al [2], 2022	Very good	Very good	Very good	Very good	Very good	Very good
Puckett [15], 2022	Adequate	Adequate	Doubtful	Doubtful	Doubtful	Doubtful
Addeo et al [16], 2023	Adequate	Adequate	Doubtful	Doubtful	Doubtful	Doubtful

a PROM: patient-reported outcome measure.

The studies by van Deursen et al [14] and van Deursen et al [2] received a “very good” final rating, having systematically met the COSMIN criteria, including a well-designed and adequately reported pilot phase. In contrast, the studies by Puckett [15] and Addeo et al [16] showed limitations in the implementation and reporting of comprehensibility testing and cognitive interviews, leading to reduced methodological transparency and a final rating of “adequate.”

The quality of the psychometric properties of the instruments was assessed according to the criteria proposed by Terwee et al [13], classifying each property as sufficient (+), insufficient (–), or indeterminate (?; Table 5). Regarding reliability, all 4 studies reported adequate levels, with Cronbach α coefficients exceeding 0.70, indicating sufficient internal consistency (+). Structural validity was also rated positively in all cases as EFA and/or CFA were applied, thus meeting the established standards (+).

**Table 5.** Evaluation of psychometric properties.

Study	Reliability	Structural validity	Content validity	Convergent validity	Discriminant validity
van Deursen et al [14], 2015	+^a^	+	+	?^b^	?
van Deursen et al [2], 2022	+	+	+	+	+
Puckett [15], 2022	+	+	+	+	+
Addeo et al [16], 2023	+	+	+	?	?

a Sufficient.

b Indeterminate.

In terms of content validity, all 4 instruments were grounded in solid theoretical frameworks and developed based on prior literature or expert review, leading to a sufficient rating (+). With respect to convergent and discriminant validity, only the studies by van Deursen et al [2] and Puckett [15] provided clear empirical evidence supporting these aspects of construct validity and, therefore, were rated as sufficient (+). In contrast, the studies by van Deursen et al [14] and Addeo et al [16] did not report enough information to determine the presence of these forms of validity and, thus, were rated as indeterminate (?).

Finally, none of the reviewed instruments used advanced measurement models such as Rasch analysis or IRT, which represents a limitation in terms of more rigorous psychometric evaluation.

### Summary of the Most Relevant Results

The 4 studies contributed recent, culturally contextualized instruments for measuring various dimensions of the digital divide. Notably, they reported adequate reliability levels and validated their structures through factor analysis. However, aspects such as convergent and discriminant validity, as well as the use of advanced measurement models, were less frequently addressed.

From a methodological perspective, all studies defined their theoretical content, but limitations were noted in the studies by Puckett [15] and Addeo et al [16] regarding the implementation of pilot-testing and the detailed evaluation of item comprehensibility, which may affect instrument validity in certain contexts [2,16].

Despite these limitations, the validated instruments—particularly the ISS by van Deursen et al [14] and the IoTSS by van Deursen et al [2]—represent valuable tools for analyzing digital inequality in social and health care settings. These tools may be further strengthened in future research through broader validation efforts.

## Discussion

### Principal Findings

This study is framed within the growing interest in assessing the digital divide arising from unequal access to, use of, and benefit from health technologies, particularly following the rapid digital transformation of recent years accelerated by the COVID-19 pandemic. The digital divide has been widely recognized as a social determinant that conditions equitable access to health care in increasingly digitized environments. This inequality has driven the development of measurement instruments for various contexts, along with the assessment of their corresponding psychometric properties [2,18].

Although the digital divide has 3 levels, as described in the Introduction section, the instruments identified in this review predominantly map onto the second level (digital skills and use) and third level (digital capital and tangible outcomes). Specifically, the scales by van Deursen et al [14] and Puckett [15] operationalize the transition from technical competencies (second level) to social and economic benefits (third level). This focus is crucial for health contexts as it moves beyond mere physical access (first level) to measure effective engagement with digital health resources. The review findings reveal measurement instruments for the digital divide that demonstrate adequate reliability levels and validated structures through factor analysis. However, methodological limitations persist in some studies, such as those by Puckett [15] and Addeo et al [16], including the absence of pilot-testing, inadequate evaluation of item comprehensibility, and a lack of complementary analyses—such as Rasch modeling—that could strengthen scale validity and robustness [2,16]. These gaps complicate cross-study comparisons and limit applicability across diverse contexts.

A critical issue identified is the limited cross-cultural adaptation of the analyzed instruments. None of the included studies [2,14-16] carried out formal translation or linguistic validation processes for use beyond their original context, which restricts their cultural applicability and relevance to Spanish-speaking populations. Nevertheless, the ISS [14] and IoTSS [2] evaluate general competencies, facilitating potential cross-cultural adaptation.

The IoTSS by van Deursen et al [2] stands out as one of the instruments with the strongest psychometric properties and high applicability among young populations, with potential utility in Spanish-speaking educational and health care settings. Its use could promote international result comparisons and inform the design of more context-specific interventions [2].

Adapting measurement tools for vulnerable populations represents a pressing need in public health and clinical research. Sociodemographic factors such as advanced age, migrant status, or rural residence influence access to health care services, health literacy, and psychosocial well-being [19,20]. For instance, research conducted in China by Tang et al [19] demonstrated that the digital divide negatively impacts quality of life and mental health among older adults in rural areas, primarily due to limited information seeking skills. This finding underscores the importance of culturally adapting measurement instruments by incorporating variables that reflect the social, technological, and educational conditions of diverse population groups.

Despite progress in the design of health-related measurement instruments, there is limited integration of validated scales in cross-sectional studies involving vulnerable populations. Evidence shows that phenomena such as the “grey digital divide” described by Mubarak and Suomi [21] significantly affect older adults, who face technological and social barriers that hinder access to essential services, including health care. The scarcity of cross-sectional studies using validated scales restricts the generation of comparable data and limits the planning of evidence-based interventions [21].

Nursing, due to its central role in health promotion and patient education, should lead cross-cultural validation and adaptation processes for measurement instruments in vulnerable settings. This leadership aligns with nursing practice, characterized by a holistic approach and close ties to community environments. Nurses also possess the necessary competencies to ensure that measurement tools are not only linguistically translated but also adapted to the cultural, social, and educational specificities of target populations, taking into account literacy levels, access to IT, and social support networks [19].

The COVID-19 pandemic critically highlighted structural inequalities, particularly those related to the digital divide and its impact on vulnerable groups. In education, Golden et al [22] documented how the transition to online learning amplified preexisting disparities among racialized and socioeconomically disadvantaged youth, affecting their academic performance and access to mental health resources. Similar inequities are observed in digital health care access, impacting older adults, rural residents, and migrants.

Furthermore, Meka’a et al [23] demonstrated that digital technology use significantly increased the likelihood of youth employment in a Cameroonian population, indicating that information and communications technologies can play a protective role in socioeconomic well-being. However, the lack of internet access or digital devices creates structural gaps that are difficult to overcome [23,24]. Therefore, measurement scale adaptation must consider these sociocultural variables to ensure valid and clinically useful results [22].

The limited integration of measurement instruments into intervention studies or public policies compromises the evaluation of strategies aimed at reducing the digital health divide. Without valid, reliable, and culturally adapted tools, there is a risk of perpetuating—or even exacerbating—inequities in access to digital health technologies, undermining the effectiveness of health care digital transformation [19,21]. Recent research [19,21,25] highlights that insufficient technology access and low digital literacy deepen social and health inequalities. Moreover, without the systematic application of culturally adapted scales, it becomes difficult to identify the actual barriers faced by vulnerable populations and design policies that effectively promote health equity..

### Limitations

The predominant use of cross-sectional designs in the included studies limited the assessment of temporal stability and responsiveness. Longitudinal studies are needed to evaluate how digital divide metrics evolve over time. The limited number of fully validated instruments also restricts the availability of evidence regarding the cross-cultural validity of these scales rather than the generalizability of the systematic review findings themselves [8,9]. Additionally, the limited number of studies conducted across diverse demographic or cultural contexts restricts the assessment of broader scale applicability.

### Implications for Nursing Practice

Nurses should not only apply validated instruments but also actively engage in their cultural adaptation, ensuring that assessment tools are understandable and relevant for the most vulnerable social groups. For rural populations, older adults, or communities with low digital literacy, it is essential that instruments are accessible and tailored to the cognitive, technological, and sociocultural capacities of participants, thereby addressing the impact of digital exclusion on access to health care and well-being.

In clinical practice, these instruments can serve as screening tools during nursing admission assessments. By identifying patients with low digital capital or skills, nurses can tailor education strategies (opting for nondigital alternatives or providing guided assistance for telehealth use), thereby preventing exclusion from care pathways.

### Future Research

From a practical perspective, the analyzed instruments provide a useful foundation for developing new public policies, digital literacy programs, and interventions focused on digital equity. However, their applicability in real-world settings remains challenging due to the need for further strengthening of psychometric properties, particularly sensitivity to change, external validity, and cultural adequacy.

### Conclusions

The instruments identified in this review represent a significant advancement in the structured measurement of the digital divide, showing adequate internal reliability and factorial validation. Nonetheless, a critical analysis of their psychometric properties reveals common methodological limitations such as insufficient assessment of convergent and discriminant validity, lack of pilot-testing, and absence of the use of advanced analytical models such as Rasch or IRT.

Moreover, none of the instruments have undergone cross-cultural adaptation for use outside their original linguistic contexts. Despite these limitations, the reviewed tools hold substantial potential for use in health care, particularly if their validation and adaptation processes are strengthened.

Finally, there is a clear need to expand research in this area in terms of both the number of studies and their methodological quality to develop robust instruments capable of accurately and contextually assessing the digital health divide. Future studies should prioritize validation in vulnerable groups, use advanced analytical models, and incorporate cross-cultural adaptation and uniform scale validation to enable a more precise, inclusive, and comparable evaluation of the digital divide in health.

## Supplementary material

10.2196/82005 Checklist 1 PRISMA 2020 checklist.

## Abbreviations

CFA: confirmatory factor analysis
COSMIN: Consensus-Based Standards for the Selection of Health Measurement Instruments
EFA: exploratory factor analysis
ICC: intraclass correlation coefficient
IoTSS: Internet of Things Skills Scale
IRT: item response theory
ISS: Internet Skills Scale
PRISMA: Preferred Reporting Items for Systematic Reviews and Meta-Analyses
